# Nitrous Oxid Gas and Oxygen Anesthesia

**Published:** 1899-11

**Authors:** 


### Nitrous Oxid Gas and Oxygen Anesthesia.

At a recent meeting of the Society of Anesthetists, Gardner (*Brit. Med. Jour.*, April 29, p. 1031) pointed out that short operations, such as tenotomy, catheterization, movement of stiff joints, pelvic examinations, etc., can be satisfactorily performed under the influence of nitrous oxid gas and oxygen. After the administration has lasted a few minutes, from 15 to 20 percent of oxygen is required to avoid muscular rigidity. The resulting anesthesia is less profound than that of chloroform or ether. Under it there is excitation of reflexes of adjacent limbs, etc., and reflex alteration in respiration. Retching movements and sickness can not be prevented by "pushing" this anesthetic. Florid, alcoholic and muscular men are not good subjects. The administration itself should be achieved only by the expenditure of forty gallons of the mixture over a quarter of an hour.

